# Pathogenic Leptospira Species Are Widely Disseminated among Wild Rodents in Urban Areas of Guangzhou, Southern China

**DOI:** 10.3390/microorganisms10050873

**Published:** 2022-04-22

**Authors:** Jian-Wei Shao, Yue-Hong Wei, Xin-Yan Yao, Hai-Yan Chen, Hong Liu, Jing Sun, Shou-Yi Chen

**Affiliations:** 1 School of Life Science and Engineering, Foshan University, Foshan 528225, China; jwshao1988@163.com (J.-W.S.); yaoxinyan95@163.com (X.-Y.Y.); liuhong11252021@163.com (H.L.); sj1075963621@163.com (J.S.); 2 Department of Parasitic Disease and Endemic Disease Control and Prevention, Guangzhou Center for Disease Control and Prevention, Guangzhou 510440, China; wei_yh0928@163.com (Y.-H.W.); chenhy2020@hotmail.com (H.-Y.C.); 3 Institute of Public Health, Guangzhou Medical University, Guangzhou 511436, China

**Keywords:** pathogenic *Leptospira*, southern China, urban areas, wild rodents

## Abstract

Leptospirosis is a neglected zoonotic disease with global importance caused by pathogenic *Leptospira*. Rodents are considered the most significant reservoirs for both human and animal infection. Historically, Guangzhou has been an endemic region of human leptospirosis. Although the incidence in humans has significantly decreased in the past decades in China, the epidemiology of pathogenic *Leptospira* in wild rodents is of great significance for the prevention and control of human leptospirosis. In this study, a total of 296 wild rodents were trapped in urban areas of Guangzhou, in southern China, in 2020. Three pathogenic *Leptospira* species, i.e., *Leptospira interrogans*, *L. borgpetersenii*, and *L. kirschneri*, were detected by nested PCR in this wild rodent population with an overall prevalence of 9.5%. Additionally, *L. interrogans* was detected in three of the four captured rodent species, and the relative high prevalence suggests that *L. interrogans* probably represents the preponderant species of the pathogenic *Leptospira* circulating in Guangzhou. Taken together, this study reveals a high genetic diversity of pathogenic *Leptospira* disseminated among wild rodents in the urban areas of Guangzhou and emphasizes that the risk for the occurrence of human leptospirosis in Guangzhou remains high.

## 1. Introduction

Leptospirosis is a neglected zoonotic disease of global public health concern caused by pathogenic spirochetes of the genus *Leptospira* [1,2]. The WHO reported that the global burden of leptospirosis potentially reaches up to one million severe human cases, with more than 60,000 deaths annually [3,4]. Formerly, human leptospirosis was prevalent mainly in east/southeast Asia and South America [5]. However, it has recently been recognized as an emerging or re-emerging infectious disease frequently reported in Europe, North America, and Africa [4,6,7,8]. As a result, leptospirosis has emerged globally as an important infectious disease and has attracted increasing attention around the world [2].

The genus *Leptospira* comprises highly diversified bacteria that can be divided into pathogenic, intermediate, and saprophytic groups based on their genetic characteristics [1,2,9]. The species of the pathogenic group, including *Leptospira interrogans*, *L. borgpetersenii*, *L. kirschneri*, *L. alexanderi*, *L. alstonii, L. kmetyi*, *L. noguchii*, *L. santarosai*, *L. mayottensis*, and *L. weilii*, are the major etiological agents of leptospirosis globally [1,9]. Among them, *L. interrogans*, *L. borgpetersenii*, and *L. kirschneri* are the most abundant species circulating worldwide [10] and also the predominant pathogenic species responsible for human leptospirosis in China [11].

Leptospirosis is a category B notifiable disease in China and has joined the list of nationally monitored infectious diseases since 1955 [11]. The first human leptospirosis case in mainland China was reported in the Guangdong province in 1934 and was followed by confirmed cases in the majority of provinces and areas of China, ultimately resulting in more than 2.5 million cases and over 20,000 deaths [12]. Based on different human leptospirosis incidence rates, China can be divided into four regions [12,13]. Guangzhou, the provincial capital of Guangdong and the largest city of southern China, is located in region A, which historically has had a relatively high incidence rate [12,13]. Thanks to the national surveillance system and prevention and control programs, improved housing, better waste management, and the improvement of agricultural mechanization, the average annual incidence of human leptospirosis in China has dramatically decreased [13]. A recent study indicated that for the past decade, human leptospirosis in China was primarily distributed in central-southern China, including the Guangdong province [14]. According to data available from the official departments of mainland China, the total number of confirmed leptospirosis cases nationwide was 297 in 2020, of which 23 cases were reported in the Guangdong province (http://www.nhc.gov.cn/jkj/s3578/202103/f1a448b7df7d4760976fea6d55834966.shtml, accessed on 12 March 2021; http://wsjkw.gd.gov.cn/zwgk_zwwgk_jggk/content/post_3227857.html, accessed on 19 February 2021).

At least 200 species of animals, including mammals, birds, amphibians, and reptiles, have been identified as natural carriers of pathogenic *Leptospira* species worldwide [9,15,16]. In China, more than 60 species of wild and domestic animals are confirmed hosts [12]. Rodents are considered prominent maintenance hosts for *Leptospira* spp. and may transfer the infection to livestock, pets, and humans [17]. As a result, the surveillance of pathogenic *Leptospira* in rodents is of great importance for the prevention and control of the disease. Therefore, we screened rodents sampled from urban areas in Guangzhou city for pathogenic *Leptospira* species to better understand the epidemiology of these bacteria in wild rodents.

## 2. Materials and Methods

### 2.1. Rodents Trapping and Samples Processing

**Figure 1:**
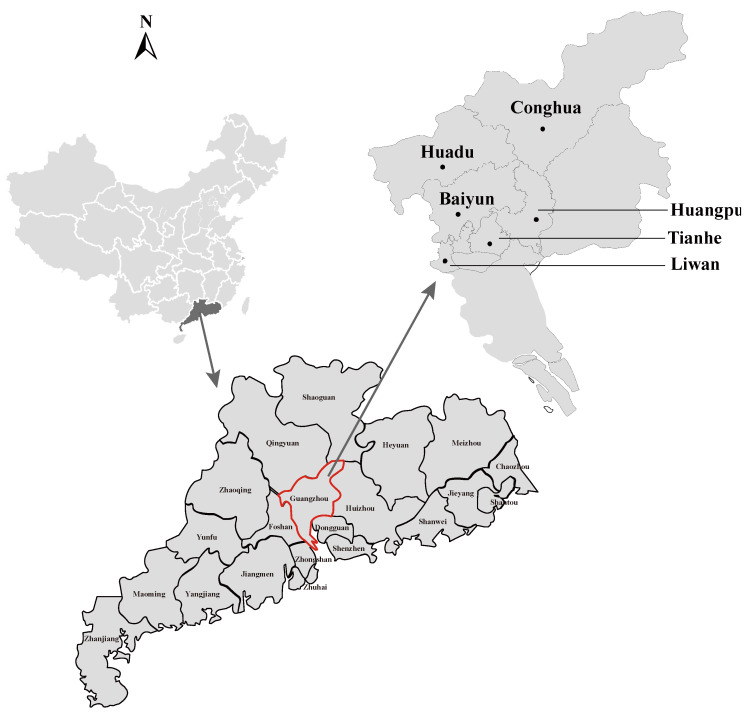
Geographic maps showing the location of sampling sites from where the rodents were captured in this study. This map was plotted using a combination of Surfer software, Version-4 (Golden Software, Golden, CO, USA), and Adobe illustrator, Version CC2017 (Adobe, San Jose, CA, USA). The black dots indicate the sampling regions in this study.

Rodents were trapped in cages using cooked food as bait in the urban areas of six districts (Liwan, Huadu, Tianhe, Huangpu, Baiyun, and Conghua) in Guangzhou city in 2020 (Figure 1). Specifically, rodents were captured in the inner-city districts of Liwan, Huadu, Tianhe, Huangpu, and Baiyun, and in the sub-urban area of Conghua. The rodent species, weight, age, and sex were identified through morphological examination performed by trained field biologists, and the species were further confirmed by sequence analysis of the mt-*cyt* b gene [18]. All rodents were captured alive and killed immediately after anesthesia. The kidney tissue samples were collected and stored at −80 °C until further use.

Approximately 30 mg of kidney samples were homogenized in 500 μL sterile phosphate buffered saline solution (PBS, pH = 7.02, GIBCO), and the total DNA was extracted using a DNA extraction kit (OMEGA, Doraville, CA, USA), according to the manufacturer’s instructions. Then, all extracted DNA samples were screened for the presence of DNA from pathogenic *Leptospira*.

### 2.2. Detection and Molecular Characterization of Pathogenic Leptospira

*Leptospira* was detected using a nested PCR, targeting a conserve region of the 16S rRNA (*rrs*) gene in pathogenic *Leptospira* species [19]. To better identify and characterize the different species in the positive samples, the nearly complete sequences of the *rrs* gene and the sequence of the *secY* gene, which has shown to be suitable for phylogenetic analysis of pathogenic *Leptospira* [20,21], were also recovered. Distilled water was used as a negative control for PCR amplification. The primer sequences used in this study are listed in Table 1.

The PCR products of the expected size, according to each set of primers, were purified using a gel extraction kit (TaKaRa, Dalian, China) after electrophoresis. The purified DNA was cloned into a pMD19-T vector (TaKaRa, China), and the resulting plasmid was used to transform competent *E. coli* cells. Positive inserts were confirmed by PCR, and five positive clones were sequenced by the Sangon Biotechnology Company (Shanghai, China). To prevent contamination, the preparation of the PCR mix and the addition of the template DNA were performed in separate rooms using dedicated pipets and filtered tips.

### 2.3. Sequence Comparison and Phylogenetic Analysis

Sequence assembly and manual editing were performed using the SeqMan program (DNASTAR, Madison, WI, USA). The nucleotide (nt) sequence identities were calculated by the MegAlign program available within the Lasergene software package (version 7.1, DNAstar) [22]. All the sequences obtained in this study have been submitted to GenBank under the accession numbers OK617223–OK617250 and OK632480–OK632507.

The best-fit evolutionary model of nt substitution was determined using jModelTest [23]. The maximum-likelihood (ML) trees were constructed based on the general time-reversible (GTR) nucleotide substitution model and the optimized parameters of gamma (Γ)-distribution and proportion of invariable sites (i.e., GTR + Γ + I) with bootstrap support values calculated from 100 replicates implemented in MEGA X [24]. All phylogenetic trees were mid-point rooted for purposes of clarity only.

### 2.4. Statistical Data Analysis

The statistical analysis was performed using the Statistical Package for Social Sciences Version 22.0 software (SPSS, Chicago, IL, USA). Chi-square tests were performed to determine differences of positive rates between different Leptospira species. A *p*-value < 0.05 was considered statistically significant. The 95% confidence interval was calculated using the Epitools (https://epitools.ausvet.com.au/trueprevalence, accessed on 1 December 2021), with the parameters listed as follows: sensitivity, 0.9; specificity, 0.99; confidence interval, 95%; confidence intervals for apparent prevalence, Wilson.

### 2.5. Ethics Statement

The authors confirm that the study complies with the ethical policies of the journal, as specified in the journal’s guidelines. The sampling procedures and sample processing were approved by the ethics committee of the Guangzhou Center for Disease Control and Prevention. All animals were treated in strict accordance with the Rules for the Implementation of Laboratory Animal Medicine (1998) from the Ministry of Health, China.

## 3. Results

### 3.1. Detection of Pathogenic Leptospira

A total of 296 rodents, including 250 *Rattus norvegicus*, 40 *R. losea*, 2 *R. tanezumi*, and 4 *Mus musculus*, were captured in 6 districts of Guangzhou city (Figure 1 and Table 2). Among all rodents, the female and male rodents numbered 123 and 173, respectively. In addition,272 rodents were adult, and only 24 rodents were juveniles. After screening the total DNAs extracted from the kidneys of these rodents using primers targeting the *rrs* gene of pathogenic *Leptospira*, PCR products of expected sizes were detected in 28 samples. Overall, the mean positive rate was 9.5% (Table 2), and *R*. *norvegicus* and *R. losea* showed relatively high infection rates of 8.8% and 12.5%, respectively. The positive rate of pathogenic *Leptospira* infection in *Mus musculus* was 25.0%, which was higher than that in other rodent species. However, this difference was not statistically supported due to the limited number of *Mus musculus* collected. Importantly, the infection rates of pathogenic *Leptospira* in *R. norvegicus*, which were captured in all sampling sites, ranged from 4.2% to 30%, with the highest prevalence in the *R. norvegicus* captured in the Conghua district.

### 3.2. Molecular Characterization of Pathogenic Leptospira

To better identify and characterize the detected pathogenic *Leptospira* species, the nearly complete *rrs* and *secY* gene sequences were recovered. Sequencing and analyses using the Basic Local Alignment Search Tool (BLAST) based on the *rrs* gene sequences (1100 bp) revealed that all *Leptospira* strains detected in this study were determined as *L. interrogans* (*n* = 17), *L. borgpetersenii* (*n* = 9), and *L. kirschneri* (*n* = 2).

The *rrs* and *secY* gene sequences of *L. interrogans* recovered from the 17 positive samples of this study were closely related to each other, with 99.6–100.0% and 98.3–100.0% of nt homology, respectively, and also shared high nt homology with the corresponding gene sequences of *L. interrogans* retrieved from GenBank (99.7–100.0% for *rrs* gene sequences and 97.3–99.9% for *secY* gene sequences). Similarly, the *rrs* and *secY* gene sequences of *L. borgpetersenii* obtained in this study shared, respectively, 99.2–100.0% and 97.9–100.0% nt homology with each other and exhibited 99.3–100.0% and 97.7–99.7% nt homology with other known *rrs* and *secY* gene sequences. Meanwhile, the *rrs* and *secY* gene sequences from the two *L. kirschneri* strains obtained herein shared 99.9% and 99.3% nt homology with each other, respectively, and 99.6–100.0% and 97.2–99.8% nt homology for the *rrs* and *secY* gene in blast analysis.

**Figure 2:**
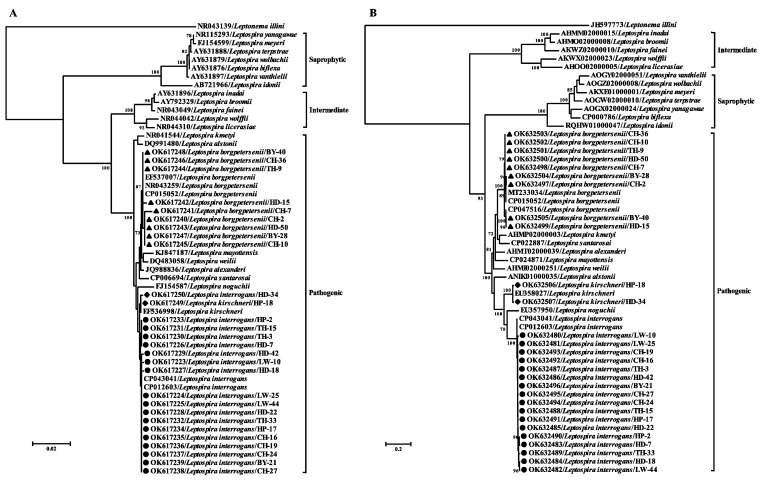
Maximum-likelihood phylogenetic trees based on the nucleotide sequences of (**A**) *rrs* and (**B**) *secY* gene of pathogenic *Leptospira*. Bootstrap values were calculated with 1000 replicates of the alignment, and only bootstrap values >70% are shown at appropriate nodes. The trees were mid-point rooted for clarity only, and corresponding sequences of *Leptonema illini* were used as the ourgroup. Sequences of *Leptospira* determined in this study are shown with different labels.

The reconstructed phylogenetic trees based on the *rrs* and *secY* sequences showed that all the *Leptospira* strains identified in this study fell into the pathogenic group and were clearly divided into three clades and clustered together with *L. interrogans*, *L. borgpetersenii*, and *L. kirschneri*, respectively (Figure 2).

### 3.3. Distribution of Pathogenic Leptospira in Rodents

The genetic and phylogenetic analyses revealed that three pathogenic *Leptospira* species (*L. interrogans*, *L. borgpetersenii*, and *L. kirschneri*) were present in the sampled population. Specifically, *L. interrogans* was detected in three out of the four captured rodent species, excluding *R. tanezumi*, while *L. borgpetersenii* was detected in *R. norvegicus* and *R. losea*, and *L. kirschneri* was only detected in *R. norvegicus*. These three pathogenic species were simultaneously detected in *R. norvegicus* (Table 3). Additionally, the overall positive rate of *L. interrogans*, *L. borgpetersenii*, and *L. kirschneri* among all captured rodents was 5.7% (17/296), 3.0% (9/296), and 0.7% (2/296), respectively (Table 3). A significant difference was observed among the positive rate of these three pathogenic species in rodents (χ^2^ = 12.464; *p* = 0.02). Among the 28 *Leptospira* strains detected in the present study, *L. interrogans* (60.7%, 17/28) was the predominant species, followed by *L. borgpetersenii* (32.1%, 9/28) and *L. kirschneri* (7.1%, 2/28).

## 4. Discussion

China has a complex eco-geography and climate, and, in most areas, natural conditions are suitable for the survival of pathogenic *Leptospira* and their host animals. As a consequence, leptospirosis is a common and widespread zoonotic disease in China [11,12]. Although historically Guangdong province has had a relatively high incidence of human leptospirosis, in recent years, the disease has been only sporadically prevalent in this region [12,13,14]. However, limited information is available regarding the epidemiology of pathogenic *Leptospira* in wild rodents, which are the most important natural reservoirs for this pathogen. Even with limited number of captured rodents within few sampling areas, this study indicates that pathogenic *Leptospira* is disseminated among wild rodents in southern China, where this disease has historically had a relatively high incidence rate. Notably, this study identified *L. interrogans*, *L. borgpetersenii*, and *L. kirschneri*, which represent the most abundant species circulating worldwide [10] and are the predominant pathogenic *Leptospira* species responsible for human leptospirosis in China [11], suggesting the potential risk of *Leptospira* transmission from wild rodents to humans Guangzhou.

In this study, *L. interrogans* was detected in three out of four rodent species with the highest prevalence among the three detected *Leptospira* species. This predominance could be explained by previous experimental and genomic studies, showing that a larger genome, increased numbers of environmental sensing genes, and a better capacity to regulate protein expression may provide *L. interrogans* with a strong ability to survive outside reservoir hosts [25,26]. More importantly, historically, *L. interrogans* has been the preponderance species in China and was responsible for at least 60% of the human cases of leptospirosis [12]. Previous studies also demonstrated that *L. interrogans* was the most prevalent species circulating in small wild animals in China [27,28,29]. Its high prevalence and wide distribution in rodents suggests that *L. interrogans* is probably still the predominant species of pathogenic *Leptospira* circulating in nature.

It is widely accepted that rodents play crucial roles as the hosts of pathogenic *Leptospira* and in the transmission of leptospirosis from animals to humans, owing to their high numbers and close association with human habitats [11,12]. Among all rodent species, *R. norvegicus* is known as the primary source of *Leptospira* infection in humans [1,30,31,32]. Many studies have reported the high prevalence of *Leptospira* in *R. norvegicus* worldwide [27,28,32,33,34]. In the present study, the relatively high prevalence of *Leptospira* in *R. norvegicus* captured in urban areas of Guangzhou is consistent with results from previous studies conducted in urban areas [32,35,36,37]. Collectively, these results suggest that *R. norvegicus* plays an important role in the transmission of *Leptospira* in urban environments.

With the acceleration of urbanization, more and more people live in urban ecosystems and the occurrence of urban leptospirosis is rising [38]. As an international metropolis, Guangzhou has a high population density, which is conducive to high prevalence of infectious diseases, including leptospirosis. Moreover, Guangzhou is located in tropical and subtropical regions, with a warm and wet climate [39]. The higher the population density in urban areas, the higher the chance of accessing water contaminated with *Leptospira*, especially since floods caused by climate change occur frequently, thus the high *Leptospira* prevalence in rodents indicates there is a high risk of a human leptospirosis outbreak in Guangzhou.

In the present study, a limited number of rodents were sampled in each area, and the species of the rodents were also very common, which may affect the determination of the prevalence of *Leptospira* in rodents. However, these results, although based on these limited samples, also provide the epidemiological data concerning the *Leptospira* species disseminated among rodents in the urban areas of Guangzhou. In conclusion, the present study reveals three pathogenic *Leptospira* species disseminated among wild rodents in the urban areas of Guangzhou, in southern China. The relatively high infection rate of pathogenic *Leptospira* in rodents is a reminder that a high risk for the occurrence of human leptospirosis in Guangzhou exists.

## Figures and Tables

**Table 1:** Primers used in this study.

Gene	Primers	Sequences (5′ → 3′)	Amplicon (bp)	Reference
*rrs*	nest-1F	GGCGGCGCGTCTTAAACATG	525	[19]
	nest-1R	GTCCGCCTACGCACCCTTTACG		[19]
	nest-2F	CAAGTCAAGCGGAGTAGCAA	289	[19]
	nest-2R	CTTAACCTGCTGCCTCCCGTA		[19]
	nest-1F	GGCGGCGCGTCTTAAACATG	1300	[19]
	*rrs*-1R	GTACAAGGTCCGGGAACGTA		This study
	nest-2F	CAAGTCAAGCGGAGTAGCAA	1100	[19]
	*rrs*-2R	GCGAGTTGGCTACCCTTTGT		This study
*secY*	*secY*-1F	GAAGGWCTTCTCGGAATGGTGG	1200	This study
	*secY*-1R	CCKTCCCTTAATTTTAGACTTCTTC		This study
	*secY*-2F	GCKCTYGGRATYATGCCTTA	1100	This study
	*secY*-2R	TTCATRAAGCCTTCRTAATTTCTCA		This study

**Table 2:** Prevalence of pathogenic *Leptospira* in rodents by species and location in Guangzhou.

Species	Location						Total (%,CI)
	Liwan	Huadu	Tianhe	Huangpu	Baiyun	Conghua	
*R. norvegicus*	3/46	7/50	4/50	2/48	3/46	3/10	22/250 8.8, 5.9−13.0)
*R. losea*	−	−	−	−	−	5/40	5/40 (12.5, 5.5−26.1)
*R. tanezumi*	0/2	−	−	−	−	−	0/2 (0)
*Mus musculus*	0/2	−	−	1/2	−	−	1/4 (25.0, 4.6−69.9)
Total (%,CI)	3/50 (6.0, 2.1−16.2)	7/50 (14.0, 7.0−26.2)	4/50 (8.0, 3.2−18.8)	3/50 (6.0, 2.1−16.2)	3/46 (6.5, 2.2−17.5)	8/50 (16.0, 8.3−28.5)	28/296 (9.5, 6.6−13.3)

*Leptospira* spp. DNA positive specimens/total specimens; ‘‘−’’ indicates that no animals were captured; CI: 95% confidence interval for apparent prevalence.

**Table 3:** Distribution of pathogenic Leptospira species among rodents.

Species	No. of Individuals	No. per Species (Positive Rate per Species, %, CI)		
		*L*. *interrogans*	*L*. *borgpetersenii*	*L*. *kirschneri*
*R. norvegicus*	250	12 (4.8, 2.8−8.2)	8 (3.2, 1.6−6.2)	2 (0.8, 0.2−2.9)
*R. losea*	40	4 (10.0, 4.0−23.0)	1 (2.5, 0.4−12.9)	−
*R. tanezumi*	2	−	−	−
*Mus musculus*	4	1 (25.0, 4.6−69.9)	−	−
Total (%, CI)	296	17 (5.7, 3.6−9.0)	9 (3.0, 1.6−5.7)	2 (0.7, 0.2−2.4)

‘‘−’’ indicates that no positive samples were detected; CI: 95% confidence interval for apparent prevalence.

## Data Availability

The sequences obtained in this study have been submitted to GenBank under the accession numbers OK617223–OK617250 and OK632480–OK632507.
